# Supplementary material for: AC2P20 selectively kills Mycobacterium tuberculosis at acidic pH by depleting free thiols
Source: RSC Adv. 2021 Jun 4;11(33):20089–100. doi: 10.1039/d1ra03181c (PMC8176622; doi:10.1039/d1ra03181c)
Supplement: RA-011-D1RA03181C-s001 [file RA-011-D1RA03181C-s001.pdf]

Electronic Supplementary Material (ESI) for RSC Advances.
This journal is © The Royal Society of Chemistry 2021

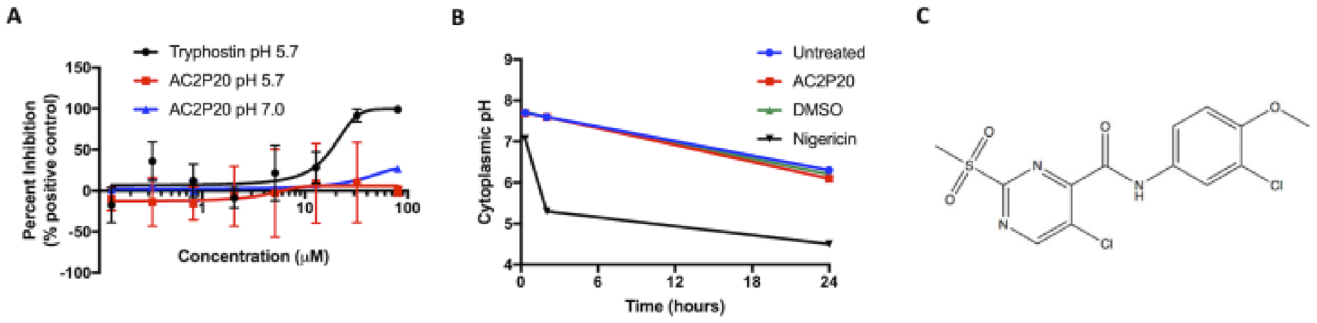

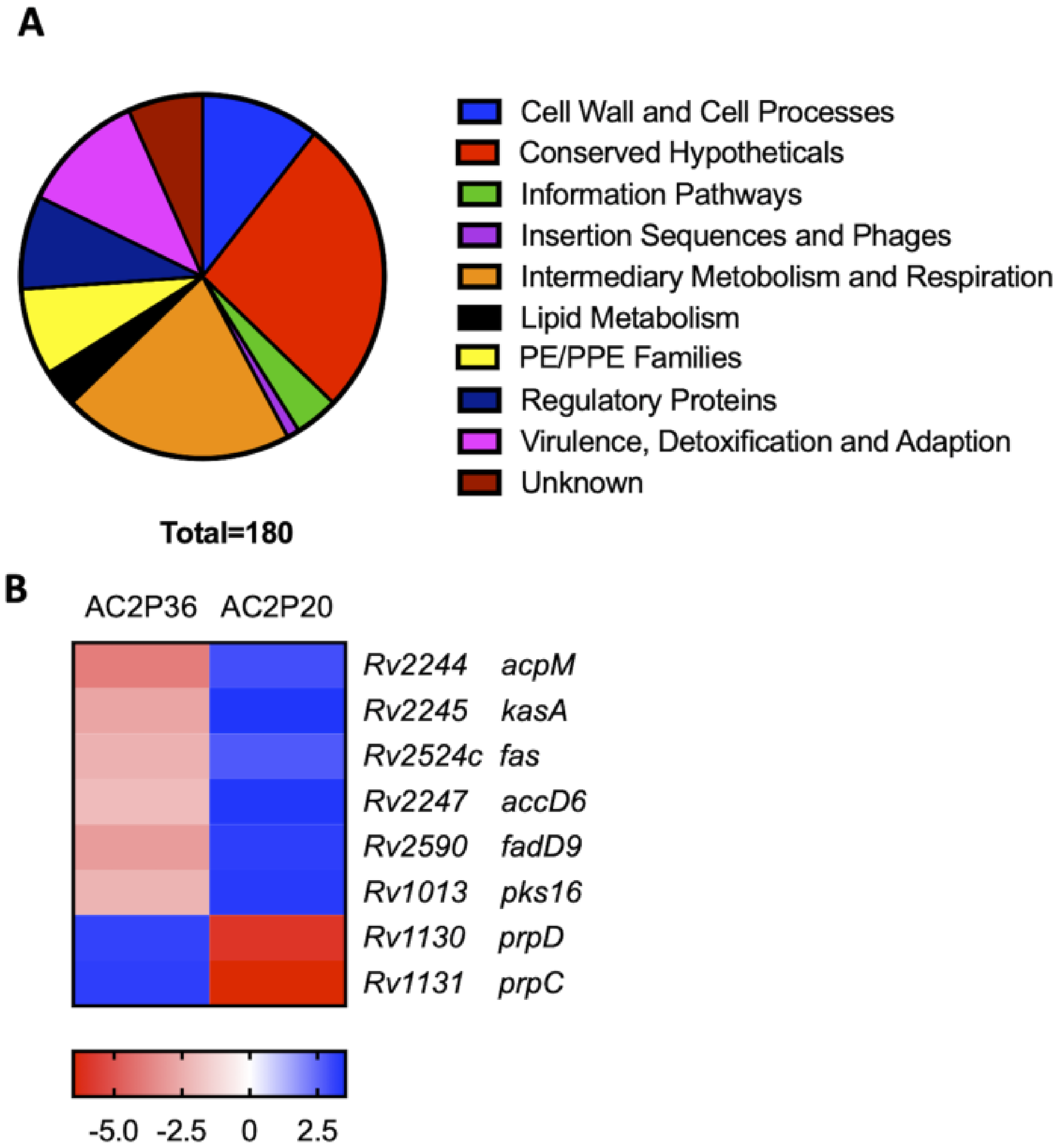
A
Cell Wall and Cell Processes
Conserved Hypotheticals
Information Pathways
Insertion Sequences and Phages
Intermediary Metobolism and Respiration
Lipid Metabolism
PE/PPE Families
Regulatory Proteins
Virulence, Detoxification and Adaption
Unknown
Total=180
B
AC2P36
AC2P20
Rv2244 acpM
Rv2245 kasA
Rv2524c fas
Rv2247 accD6
Rv2590 fadD9
Rv1013 pks16
Rv1130 prpD
Rv1131 prpC
-5.0
-2.5
0
2.5

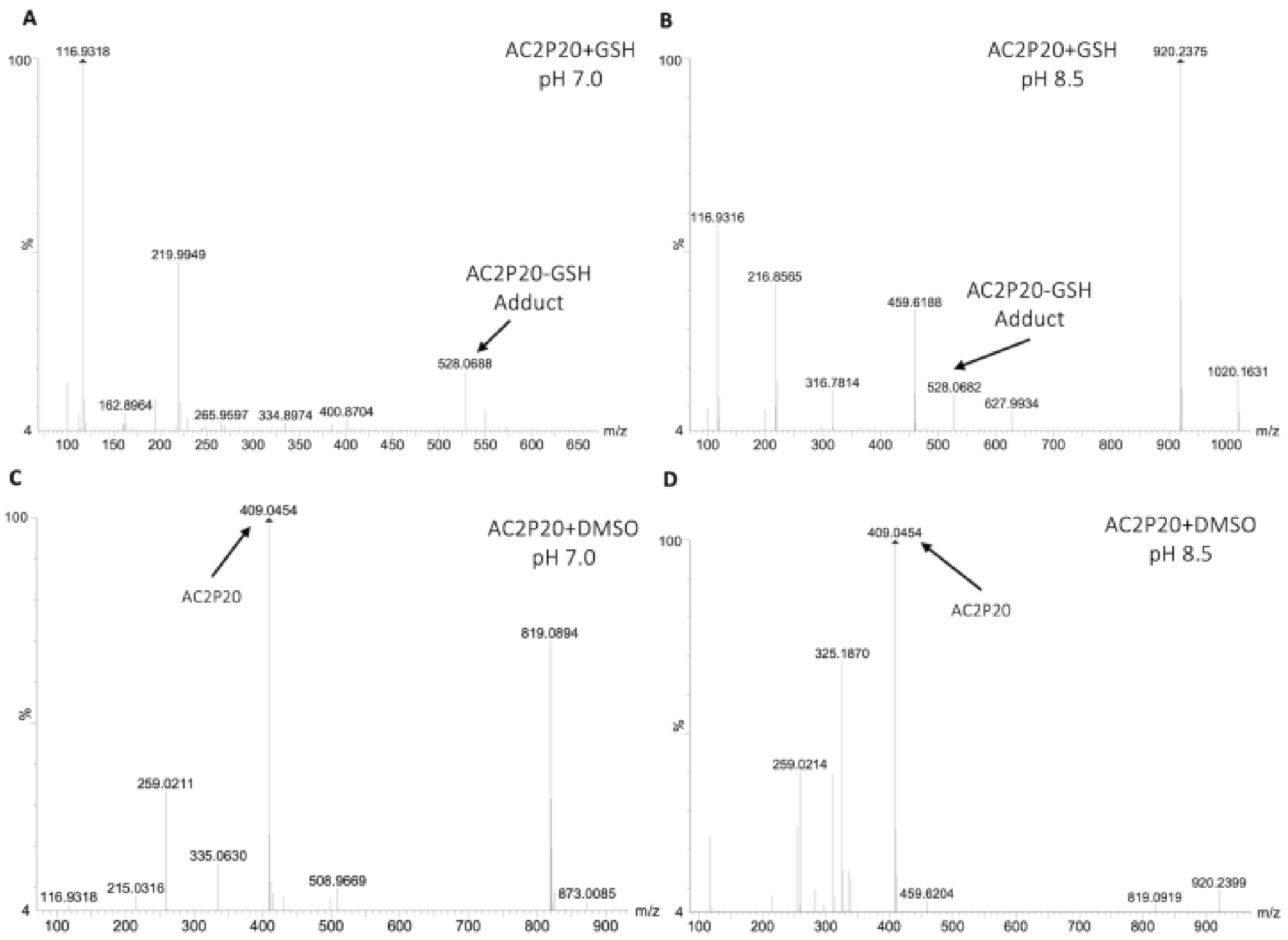
A
AC2P20+GSH
pH 7.0
116.9318
219.9949
AC2P20-GSH
Adduct
528.0688
162.8964
265.9597
334.8974
400.8704
m/z
B
AC2P20+GSH
pH 8.5
920.2375
116.9316
216.8565
459.6188
AC2P20-GSH
Adduct
316.7814
528.0682
627.9934
1020.1631
m/z
C
AC2P20+DMSO
pH 7.0
409.0454
AC2P20
819.0894
259.0211
335.0630
116.9318
215.0316
508.9669
873.0085
m/z
D
AC2P20+DMSO
pH 8.5
409.0454
AC2P20
325.1870
259.0214
459.6204
819.0919
920.2399
m/z

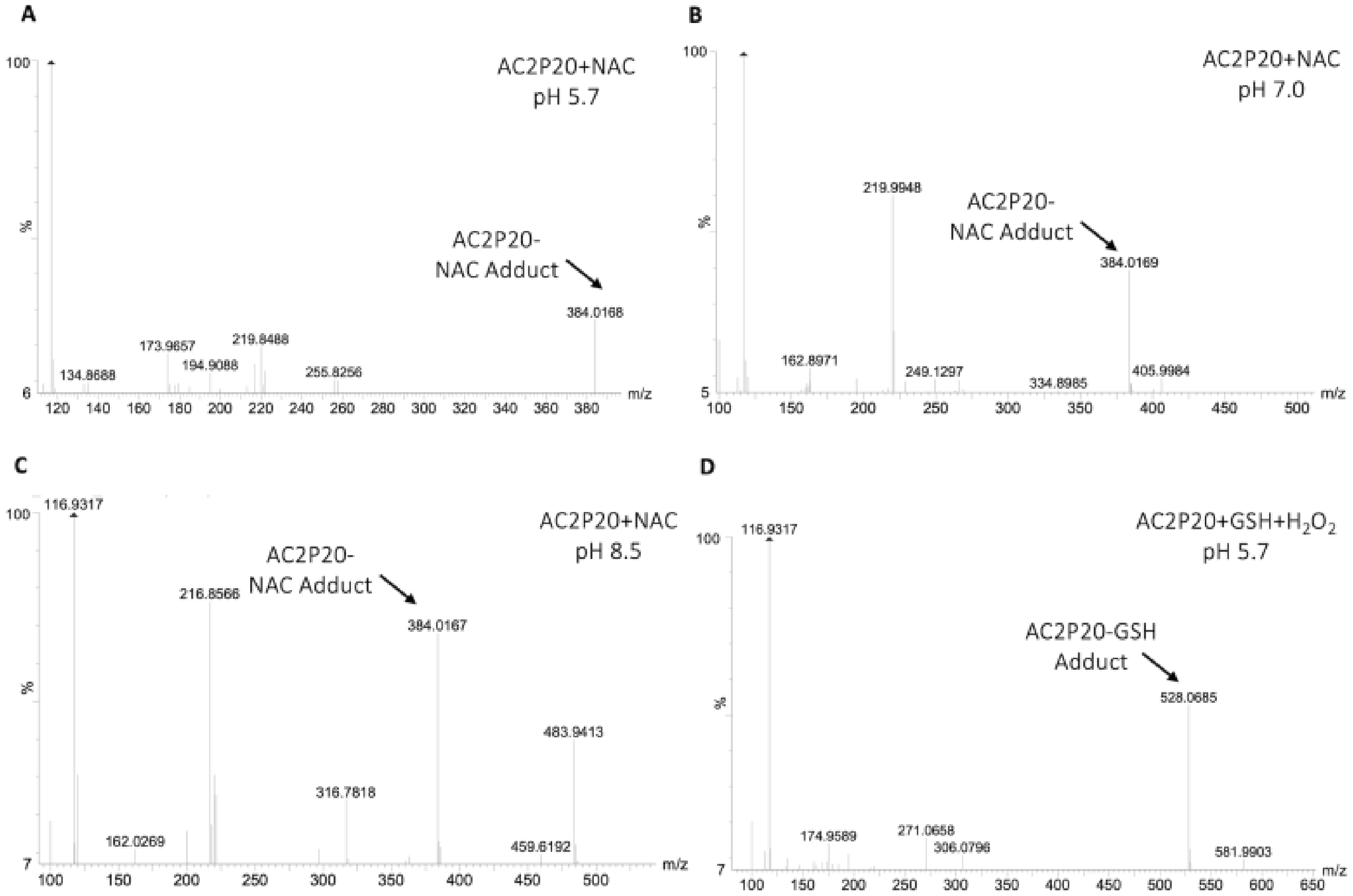
A
AC2P20+NAC
pH 5.7
AC2P20-
NAC Adduct
384.0168
173.9657
219.8488
194.9088
134.8688
255.8256
%
m/z
B
AC2P20+NAC
pH 7.0
219.9948
AC2P20-
NAC Adduct
384.0169
162.8971
249.1297
334.8985
405.9984
%
m/z
C
116.9317
AC2P20+NAC
pH 8.5
AC2P20-
NAC Adduct
216.8566
384.0167
483.9413
316.7818
162.0269
459.6192
%
m/z
D
116.9317
AC2P20+GSH+H_2O_2
pH 5.7
AC2P20-GSH
Adduct
528.0685
174.9589
271.0658
306.0796
581.9903
%
m/z

Peak (Da)	Possible Chemical Scaffold	Figure No.
130.16	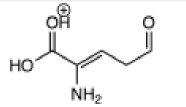	3C
178.12	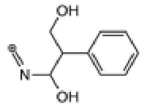	3C
194.12	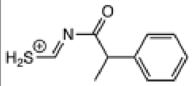	3C
206.15	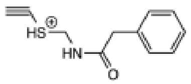	3C
222.11	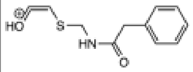	3C
384.02	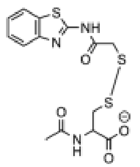	S3A, S3B, S3C
391.28	Phthalate Plasticizer	3C
401.26	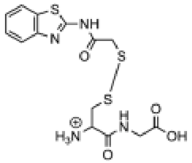	3C
409.04, 409.05	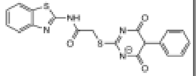	3B, S2C, S2D,
528.06, 528.07	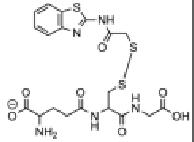	3A, S2A, S2B, S3D
530.08	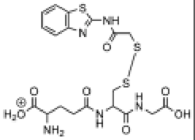	3C
