# Supplementary material for: AC2P20 selectively kills Mycobacterium tuberculosis at acidic pH by depleting free thiols
Source: RSC Adv. 2021 Jun 4;11(33):20089–100. doi: 10.1039/d1ra03181c (PMC8176622; doi:10.1039/d1ra03181c)
Supplement: RA-011-D1RA03181C-s002 [file RA-011-D1RA03181C-s002.pdf]

Electronic Supplementary Material (ESI) for RSC Advances.
This journal is © The Royal Society of Chemistry 2021

Supplemental Figures:

S1A. Dose-response curve for AC2P20 inhibition of *M. smegmatis* GFP fluorescence.

S1B. AC2P20 does not modulate Mtb cytoplasmic pH at pH 5.7. DMSO and Nigericin served as negative and positive controls, respectively.

S1C. The chemical structure of AC2P36 (5-chloro-N-(3-chloro-4-methoxyphenyl)-2-methylsulfonylpyrimidine-4-carboxamide) (29).

S2A. A pie chart depicting the functional classification breakdown of significantly induced genes (>2-fold, q < 0.05) following the analysis of AC2P36-treated Mtb RNA-seq profile.

S2B. Heatmap comparing the contrast between 8 differentially-regulated genes (between AC2P20 and AC2P36 at pH 5.7) that are involved in lipid metabolism and central metabolism. Genes were annotated with the H37Rv genome.

S3A. Mass spectrometry data showing adduct formation between AC2P20 and GSH at pH 7.0. Spectra were analyzed in negative ESI mode.

S3B. Mass spectrometry data showing adduct formation between AC2P20 and GSH at pH 8.5. Spectra were analyzed in negative ESI mode.

S3C. AC2P20 incubated with DMSO does not fragment in the absence of GSH at pH 7.0. Spectra were analyzed in negative ESI mode.

S3D. AC2P20 incubated with DMSO does not fragment in the absence of GSH at pH 8.5. Spectra were analyzed in negative ESI mode.

S4A. Mass spectrometry data showing adduct formation between AC2P20 and N-acetylcysteine at pH 5.7. Spectra were analyzed in negative ESI mode.

S4B. Mass spectrometry data showing adduct formation between AC2P20 and N-acetylcysteine at pH 7.0. Spectra were analyzed in negative ESI mode. 20 +NAC 7.0

S4C. Mass spectrometry data showing adduct formation between AC2P20 and N-acetylcysteine at pH 8.5. Spectra were analyzed in negative ESI mode.

S4D. AC2P20 is still able to form an adduct with GSH in the presence of the oxidant, H_2O_2. Spectra were analyzed in negative ESI mode.

S5. A list of labeled mass spectrometry peaks with their corresponding hypothetical chemical scaffolds.

Supplemental Table:

Table S1: Analyzed RNA-seq transcriptional profiling data. S1A. Classification of differentially regulated genes. S1B. Genes significantly induced by AC2P20. S1C. Genes significantly repressed by AC2P20. S1D. Complete list of unfiltered gene expression data in response to AC2P20 treatment.
